# Draft Genome Sequence of Arctic, Heavy Metal-Resistant *Agrococcus* sp. Strain ARC_14 Isolated from Active Layer of Permafrost from Spitsbergen (Norway)

**DOI:** 10.1128/mra.00221-22

**Published:** 2022-05-18

**Authors:** Mikolaj Dziurzynski, Anna Rokowska, Adrian Gorecki, Przemyslaw Decewicz, Anna Szych, Lukasz Dziewit

**Affiliations:** a Department of Environmental Microbiology and Biotechnology, Institute of Microbiology, Faculty of Biology, University of Warsaw, Warsaw, Poland; Montana State University

## Abstract

Extreme environmental conditions observed in polar regions create a unique ecological niche for microbial life. Bacteria living under these harsh, environmental conditions exhibit specific metabolic capabilities. In this report, we present multimetal-resistant *Agrococcus* sp. strain ARC_14, isolated from soil samples collected in Spitsbergen, Svalbard, Norway.

## ANNOUNCEMENT

*Agrococcus* species are Gram-positive, nonmotile bacteria classified in the *Actinobacteria* phylum within the *Microbacteriaceae* family (1). Currently, the genus *Agrococcus* comprises 11 species, members of which were isolated from various environments. The majority of the isolates originated from cold, nutrient-limited environments.

The source of *Agrococcus* sp. strain ARC_14 was an active layer of permafrost collected in the vicinity of the Polish Polar Station Hornsund (PPSH), Spitsbergen, Svalbard, Norway. The sample was collected from soil (0 to 10 cm depth) from the patio of the PPSH. After collection, the sample was stored at −20°C until thawing and isolation. Thawed soil samples were serially diluted using 0.1% sodium pyrophosphate buffer and plated on R2A (VWR Chemicals, Radnor, PA, USA) agar plates supplemented with nystatin (50 μg/mL) (MilliporeSigma, Darmstadt, Germany) and one of the following metal salts: 7 mM NaAsO_2_ (MilliporeSigma), 220 mM Na_2_HAsO_4_·7H_2_O (MilliporeSigma), and 7 mM K_2_CrO_4_ (MilliporeSigma). The plates were incubated at 18°C for 7 days in the dark under aerobic conditions. Observed colonies were transferred onto a new plate to obtain pure isolates. All isolates were cross-checked for their resistance to other metals. Multimetal-resistant strains were identified by the sequencing of their 16S rRNA genes obtained using the 27F and 1492R primer pair (2). The PCR product obtained for one of the strains showed high similarity to 16S rRNA from Agrococcus pavilionensis.

Genomic DNA of the ARC_14 strain (from liquid culture in R2A medium cultivated at 18°C) was isolated using the cetyltrimethylammonium bromide (CTAB)/lysozyme method (3). The genomic libraries were sequenced on an Illumina MiSeq instrument in 2 × 300 base pairs mode with the v3 chemistry kit (Illumina, San Diego, CA, USA) by using the Illumina TruSeq library kit (4). The sequencing was conducted in the DNA Sequencing and Oligonucleotide Synthesis Laboratory at the Polish Academy of Sciences (Warsaw). A total of 2,676,224 reads were obtained. The raw reads were quality controlled, filtered, and trimmed using fastp v0.23.0 run with correction mode and sliding window sequence trimming (5). Filtered reads were assembled using SPAdes v3.15.2 with --isolate flag (6). Raw assembly resulted in 114 contigs. Then, 108 contigs with SPAdes-derived coverage lower than 2.0 were removed. Additionally, 1 contig composed only of 128 guanine nucleotides was also removed. Annotation of the genome assembly was performed using the NCBI Prokaryotic Genome Annotation Pipeline (PGAP) v6.0 (7).

Final genome assembly of *Agrococcus* sp. ARC_14 is 3,265,363 bp long, with 70.67% G+C content. It is composed of five contigs with average coverage of 145×. Genome annotation predicted 3,107 protein-coding genes, 3 rRNAs, and 45 tRNAs. The genome was screened for the presence of metal resistance genes with BLASTp (E-value < 0.001, percent identity > 60, query coverage per High-scoring Segment Pair [HSP] > 80) using sequences downloaded from the BacMet database (8, 9). Genome annotation was further reannotated using MAISEN web service to determine the genomic context of investigated genes (10). Although the strain’s resistance to As(III), As(V), and Cr(VI) ions has been experimentally proven in this study, only one metal resistance gene (*arsB*), encoding arsenite and antimonite inner membrane efflux pumps (11), was identified.

### Data availability.

Genome assembly of *Agrococcus* sp. strain ARC_14 has been deposited in GenBank under the accession number GCA_022436485, and its corresponding Sequence Read Archive (SRA) accession number is SRX14250406.
